# The prognostic value of immunoscore in patients with colorectal cancer: A systematic review and meta‐analysis

**DOI:** 10.1002/cam4.1921

**Published:** 2018-12-21

**Authors:** Guorui Sun, Xiaoyuan Dong, Xiaolong Tang, Hui Qu, Hao Zhang, Ensheng Zhao

**Affiliations:** ^1^ Department of Gastrointestinal Surgery Qilu Hospital of Shandong University Jinan Shandong China; ^2^ Department of Hematology Qilu Hospital of Shandong University Jinan Shandong China

**Keywords:** colorectal cancer, immunoscore, meta‐analysis, prognosis

## Abstract

The tumor immune infiltrate, as recently evaluated with the immunoscore methodology, has been reported to be related to colorectal cancer (CRC) progression. Nevertheless, results varied from different studies. A meta‐analysis was conducted to solve this problem. We collected data from included studies to evaluate the prognostic role of immunoscore in CRC patients on overall survival (OS) and disease‐free survival (DFS). MEDLINE, EMBASE, and Cochrane libraries were searched through 30 June 2018. Hazard ratio (HR) with 95% confidence intervals (95% CI) was pooled using a random‐effects model for OS and a fixed‐effects model for DFS. Finally, eight studies (involving 4689 CRC cases) were identified as eligible publications. The results of the meta‐analysis showed that low immunoscore was significantly correlated with poor OS (HR = 1.74, 95% CI: 1.43‐2.13) and DFS (HR = 1.82, 95% CI: 1.64‐2.03). The findings from most subgroup analyses were consistent with those from the overall analysis. The immunoscore could be a useful prognostic marker in patients with CRC. It is necessary to evaluate immunological markers in international multicenter studies.

## 1 INTRODUCTION

Colorectal cancer (CRC) is the third most common cancer in men and the second most common cancer in women worldwide, with an estimated 1 849 518 new cases in 2018, and the second most common cause of death among cancer with an estimated 880 792 deaths.^1^

The American Joint Committee on Cancer/Union for International Cancer Control (AJCC/UICC) system, based on the tumor‐node‐metastases (TNM) classification, has been widely used for staging CRC patients. The system is of much importance but gives limited information for the prognostic benefit of the selected therapy.^2^, ^3^ Other markers for survival are needed.^2^ Recent studies revealed that tumor‐infiltrating lymphocytes (TILs) play an important role in increasing the anti‐tumor immunity against CRC^4^, ^5^ and other malignancies.^6^, ^7^, ^8^, ^9^, ^10^ However, TILs show heterogeneity at its target sites,^5^ just like other components in the tumor microenvironment. This heterogeneity makes it difficult to determine their roles.

Anitei MG^11^ provided evidence that the type, the density, and the location of immune cells within tumor samples strongly influenced the evolution of human CRCs. Thus, the adaptive immune reaction composed of T lymphocytes (CD3^+^) with cytotoxic (CD8^+^) and memory (CD45RO^+^) phenotype within the core of the tumor (CT) and the invasive margin (IM) was a highly significant parameter to predict recurrence and survival. To promote the use of this immune investigation as a routine testing for cancer classification, Galon^12^, ^13^, ^14^ established a methodology named “immunoscore” that provided a value based on the density of CD3^+^ and CD8^+^ lymphocytes in the CT and IM regions of tumors. Patients were stratified from I0 to I4 according to the “immunoscore” (“I”), based on the total number of observed high densities (CD3^+^ cells and CD8^+ ^cells in the tumor regions).^11^ For example, I0 refers to a tumor with low densities of CD3^+^ and CD8^+^ in the core of the tumor (CT) and the IM regions of the tumor (4‐Hi); I4 refers to tumors with high densities of CD3^+ ^and CD8^+ ^cells in both tumor regions. In addition, some studies have shown that the immunoscore method was better than the current TNM staging system, especially for colon cancers.^15^, ^16^ However, the evidence is restricted to I‐III stages of the cancer and the prognostic utility of immunoscore remains unclear.^13^

Therefore, we conducted the meta‐analysis to assess the effects of immunoscore on overall survival (OS) and disease‐free survival (DFS) in CRC patients.

## 2 METHODS

### 2.1 Literature search strategy

MEDLINE, EMBASE, and Cochrane libraries were searched to find relevant publications up to 30 June 2018, using the search terms “immunoscore OR immune score,” “colorectal cancer OR colorectal neoplasms OR colorectal carcinoma,” “prognostic OR prognosis OR survival,” and combinations thereof. This meta‐analysis was performed under the guidelines of PRISMA.^17^

### 2.2 Inclusion and exclusion criteria

All papers were reviewed by two authors (TXL and QH) independently. Uncertainties and discrepancies were resolved by consensus after discussing with a senior researcher (SGR). The selected studies had to meet the criteria as follows: (a) English articles; (b) pathologically diagnosed CRC patients; (c) OS or DFS of CRC as the research focus; and (d) reporting hazard ratio (HR) estimates with their corresponding 95% CI (or sufficient data to calculate of these effect measures). When studies were reported in duplication, only the study with the largest sample size and detailed information or the study that met the above criteria was included. Abstracts were excluded. Review articles and editorials were included if they contained original data.

### 2.3 Data extraction

Data extraction from each paper was performed by two authors (TXL and QH), and discrepancies were resolved by consensus. The following data were collected from include papers: first author, publication year, country, enrollment time, follow‐up time, characteristics of the study population (sample size, age, disease stage, and cancer site), and HR estimates with corresponding 95% CIs for OS or DFS HR from multivariate Cox proportional hazard analysis was preferred in the analysis.

### 2.4 Statistical analysis

The meta‐analysis was performed, and HR with corresponding 95% CI was calculated in CRC patients. The Cochran’s *Q* test^18^ was used to examined heterogeneity among studies by calculating the *P* value and quantified using the *I* ^2^ statistic. If *I* ^2^ < 50%, the fixed‐effects model (Mantel‐Haenszel method)^19^ was used to evaluate inter‐study heterogeneity. Otherwise, the random‐effects model (DerSimonian and Laird method)^20^ was used. Subgroup analyses for immunoscore and the OS or DFS in CRC patients were subsequently carried out according to the geographic region, number of patients, max follow‐up time, clinical stage, pathological type, and HR Sensitivity analysis was also conducted to assess the impact of each paper on the stability and strength of the results. Every time, a study in our meta‐analysis was excluded to check the impact of the study on the overall effect size. Besides, Begg’s funnel plot and Egger’s linear regression test were used to assess the potential impact of publication bias.

Stata/MP version 12 for Windows (StataCorp LP, College Station, TX, USA) was used for all statistical tests. A two‐tailed *P* < 0.05 was considered statistically significant.

## 3 RESULTS

### 3.1 Literature search

**Figure 1:**
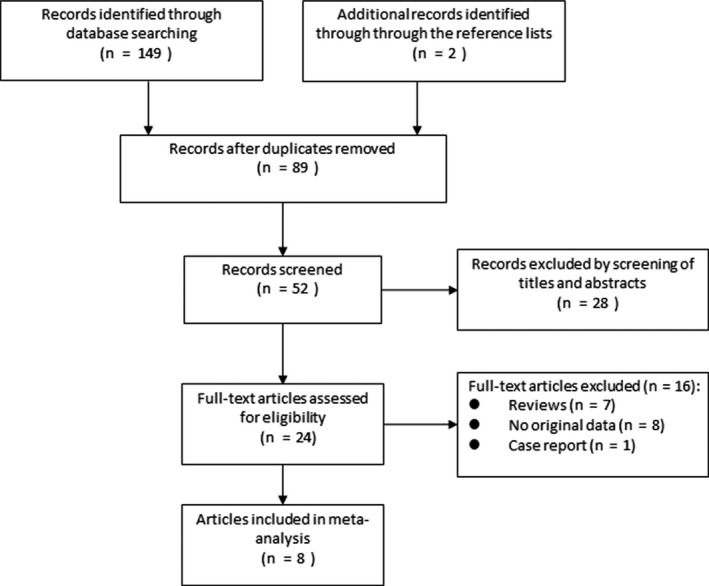
The PRISMA flow diagram of systematic literature search

Figure 1 shows the study selection process. Electronic searches identified 150 articles, of which 24 were eligible for full‐text review after screening by title and abstract. After revision, 16 of these 24 papers were excluded for following reasons: eight studies did not provide HRs or CIs, seven studies were reviews, and one study was case report. Therefore, eight studies fulfilled our eligibility criteria for the analysis.^11^, ^21^, ^22^, ^23^, ^24^, ^25^, ^26^, ^27^ Among them, one paper^24^ reported the results of two different population respectively, so it was treated as two studies for analysis.

### 3.2 Characteristics of the included studies

Table 1 presented the characteristics of the eight included studies. In all the studies, a total of 4689 CRC cases were included, published from 2011 to 2018. The number of patients included in each study varied from 60 to 2681. Among these studies, six studies focused on CRC, one focused on rectal cancer, and the other one focused on colon cancer. All eight papers investigated the prognostic role of immunoscore in OS, and six papers explored the prognostic impact of immunoscore on DFS. The median follow‐up period in each study varied from 36 to 110 months, and three studies did not provide accurate follow‐up data.

**Table 1:** Characteristics of the included studies

First author	Year	Year of recruitment	Country	Site	Stage	No. of patients	Age, y	Median follow‐up period (mo)	Survival analysis	Hazard ratio
Mlecnik B	2011	1995‐2004	France	CRC	I‐III	599	NA	NA	OS/DFS	Adjusted
Anitei MG	2014	1987‐2004	France	RC	I‐IV	111	NA	74 (0‐244)	OS/DFS	Unadjusted
Kwak Y	2016	2003‐2009	South Korea	CRC	I‐III	196	NA	37.3 (0.8‐104.6)	OS	Adjusted
Mlecnik B	2016	NA	France	CRC	I‐III	270	NA	NA	OS/DFS	Unadjusted
Park JH	2017	1997‐2008	United Kingdom	CRC	I‐III	331	70 (55‐85)	110 (60‐200)	OS/DFS	Adjusted
Liu RQ	2018	2013‐2016	China	CRC	IV	60	59 (49‐71)	36 (10‐57)	OS	Adjusted
Mlecnik B	2018	2004‐2010	Belgium	CRC	IV	441	NA	NA	OS/DFS	Adjusted
Pagès F	2018	2013‐2016	13 countries	CC	I‐III	2681	69 (60‐77)	96 (93‐100)	OS/DFS	Adjusted

CC, colon cancer; CRC, colorectal cancer; DFS, disease‐free survival; NA, not available; OS, overall survival; RC, rectal cancer.

### 3.3 Results of the meta‐analysis

#### 3.3.1 Overall survival

**Figure 2:**
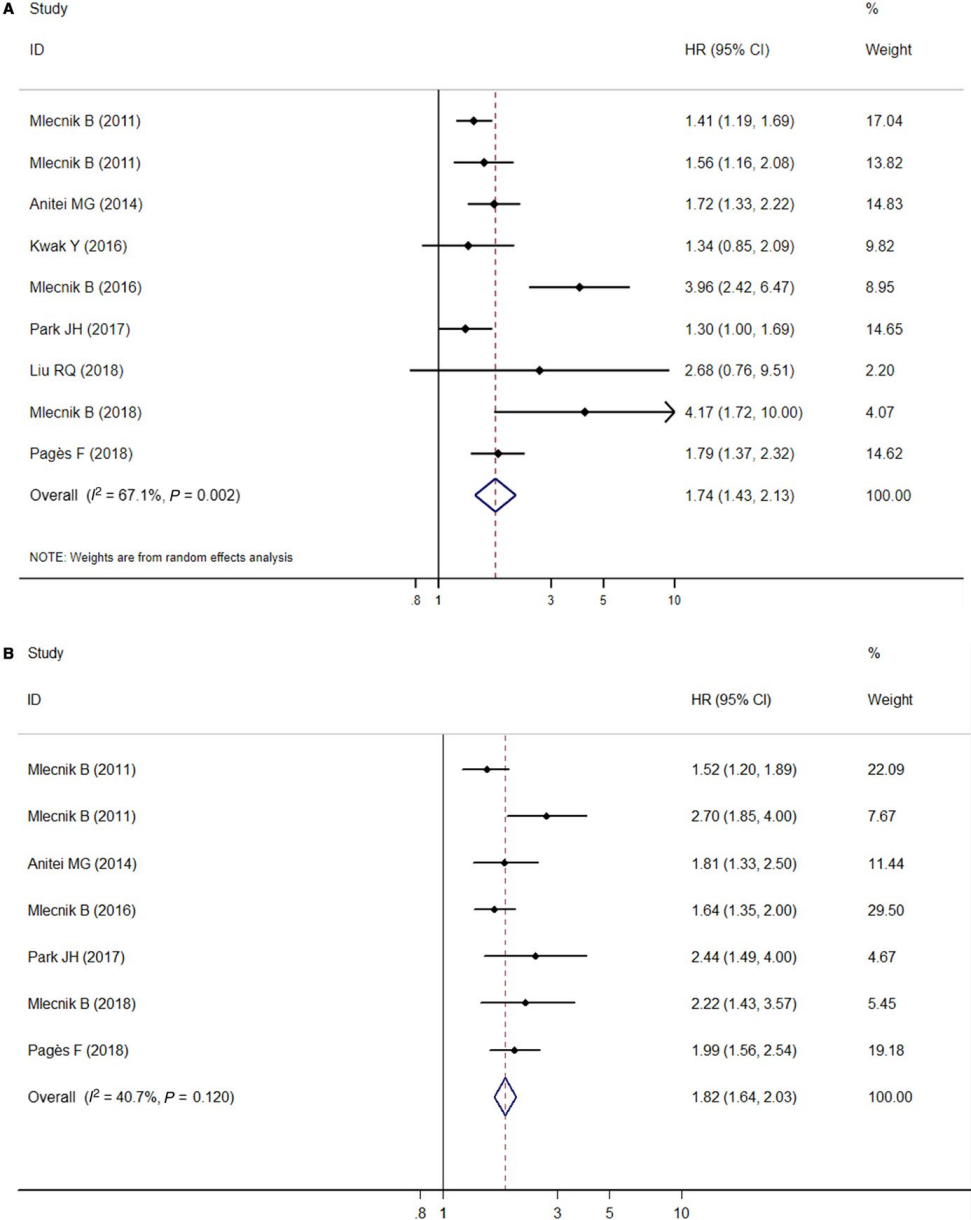
Forest plot for the association between immunoscore and colorectal cancer prognosis. Survival data are reported as overall survival (A) and disease‐free survival (B)

The heterogeneity test indicated there was moderate degree of heterogeneity among included studies; thus, a random‐effects model was employed to obtain the pooled HR. The statistical result showed that low immunoscore was significantly correlated with poor OS (HR = 1.74, 95% CI: 1.43‐2.13, *P*_heterogeneity_ = 0.002, *I* ^2^ = 67.1%) (Figure 2A).

#### 3.3.2 Disease‐free survival

The heterogeneity test indicated there was very low degree of heterogeneity among included studies; thus, a fixed‐effects model was employed to obtain the pooled HR. The statistical result showed that low immunoscore was significantly correlated with poor DFS (HR = 1.82, 95% CI: 1.64‐2.03, *P*_heterogeneity_ = 0.120, *I* ^2^ = 40.7%) (Figure 2B).

### 3.4 Subgroup analyses

Table 2 presents detailed results of subgroup analyses.

**Table 2:** Results of subgroup analyses

Group	OS				DFS			
	No. of study	HR (95% CI)	*I* ^2^, %^a^	*P* for heterogeneity test	No. of study	HR (95% CI)	*I* ^2^, %^a^	*P* for heterogeneity test
All	9	1.74 (1.43‐2.13)	67.1	<0.001	7	1.82 (1.64‐2.03)	40.7	0.120
Geographic region								
Europe	6	1.81 (1.39‐2.37)	77.5	<0.001	6	1.78 (1.58‐2.01)	47.3	0.091
Asia	2	1.46 (0.93‐2.29)	3.2	0.310	1	–	–	–
Number of patients								
<300	5	1.92 (1.37‐2.70)	64.8	0.036	3	1.82 (1.56‐2.12)	60.8	0.078
≥300	4	1.59 (1.24‐2.03)	69.2	0.011	4	1.83 (1.57‐2.12)	40.1	0.171
Max follow‐up								
<10 y	3	1.69 (1.35‐2.11)	0.0	0.418	1	–	–	–
≥10 y	2	1.50 (1.14‐1.97)	55.3	0.135	2	1.97 (1.51‐2.58)	0.0	0.318
Clinical stage								
I‐III	6	1.65 (1.31‐2.08)	72.8	0.002	5	1.80 (1.60‐2.02)	57.2	0.053
IV	2	3.61 (1.75‐7.44)	0.0	0.574	1	–	–	–
Cancer site								
CRC	7	1.80 (1.35‐2.39)	73.7	0.001	5	1.80 (1.57‐2.02)	57.8	0.050
Hazard ratio								
Unadjusted	2	2.54 (1.12‐5.74)	88.5	0.003	5	1.80 (1.60‐2.02)	57.2	0.053
Adjusted	7	1.54 (1.31‐1.81)	37.9	0.139	2	1.93 (1.49‐2.51)	0.0	0.471

CI, confidence intervals; CRC, colorectal cancer; DFS, disease‐free survival; HR, hazard ratio; OS, overall survival.

a *I* ^2^ is interpreted as the proportion of total variation across studies that are due to heterogeneity rather than chance.

The associations of immunoscore and OS in CRC patients did not differ by number of patients, max follow‐up, clinical stage, and HR When cancer cases stratified by geographic region, the low immunoscore was significantly correlated with poor OS (HR = 1.81, 95% CI: 1.39‐2.37) for Europeans, but not significantly correlated with Asians (HR = 1.46, 95% CI: 0.93‐2.29). When cancer cases stratified by clinical stage, the low immunoscore was much more significantly correlated with poor OS for IV stage patients (HR = 3.61, 95% CI: 1.75‐7.44) than for I‐III stage patients (HR = 1.65, 95% CI: 1.31‐2.08).

The associations of immunoscore and DFS in CRC patients did not differ by number of patients and HR. When cancer cases stratified by max follow‐up time, the low immunoscore was much more significantly correlated with poor DFS for more than 10 years (HR = 1.97, 95% CI: 1.51‐2.58). When cancer cases stratified by clinical stage, the low immunoscore was significantly correlated with poor DFS for I‐III stage patients (HR = 3.61, 95% CI: 1.75‐7.44), which had a similar effect for all‐stage patients.

In short, the estimated heterogeneity for studies included decreased to some degree but did not obliterate.

### 3.5 Influence analysis of individual studies

**Figure 3:**
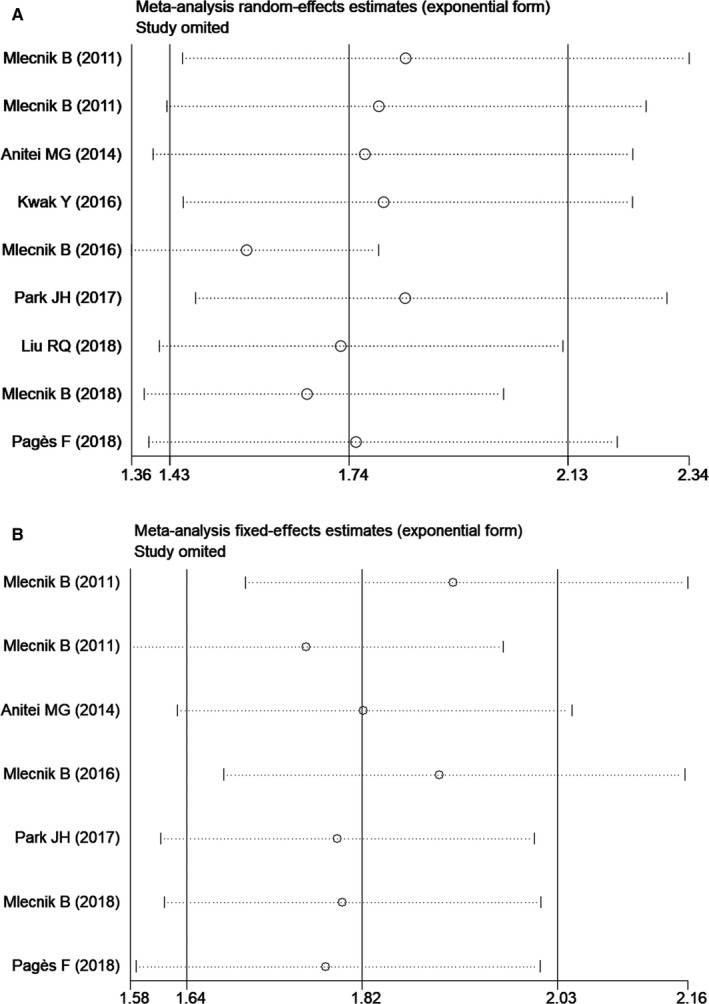
Influence analyses for omitting individual study on the summary hazard ratio. Survival data are reported as overall survival (A) and disease‐free survival (B)

To assess the impact of each single study on the pooled HR, we removed individual studies in turn from our meta‐analysis. Figure 3A,B reported the sensitivity analysis results for OS and DFS, respectively. The combined HRs for OS comparing low immunoscore to high immunoscore cancers ranged from 1.56 (95% CI: 1.36‐1.79) to 1.84 (95% CI: 1.45‐2.34). The combined HRs for DFS comparing low immunoscore to high immunoscore cancers ranged from 1.78 (95% CI: 1.57‐2.01) to 1.97 (95% CI: 1.69‐2.29). These data indicate that the combined HRs of the meta‐analysis were stable and were not overly affected by any of the eight studies.

### 3.6 Publication bias

**Figure 4:**
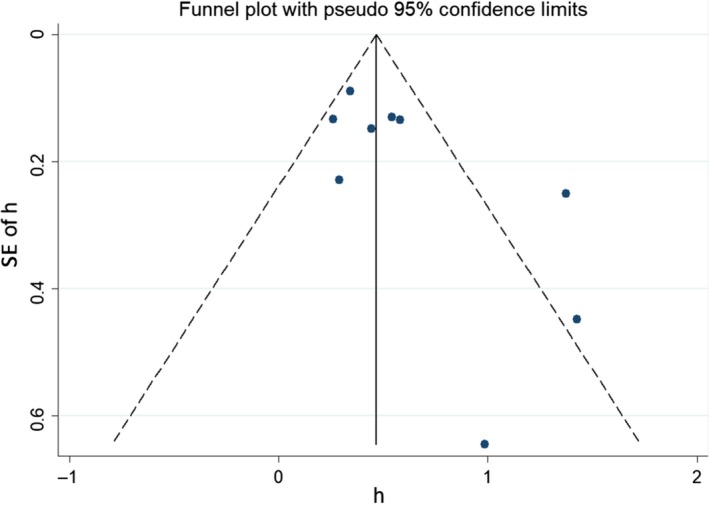
Funnel plots for publication bias of overall survival

Begg’s test (0.095), Egger’s test (0.065), and the near‐symmetric funnel plot (Figure 4) suggested that there was no significant publication bias in the meta‐analysis.

## 4 DISCUSSION

Both clinical characteristics of patients and biological features of the tumor are increasingly used and may be very helpful in the management of cancer disease. This systematic review and meta‐analysis is the first one investigating the effects of immunoscore on OS in CRCs. Our study demonstrated that the immunoscore, an immunohistochemistry‐based assessment of CD3^+^ and CD8^+^ T‐lymphocyte density, significantly correlated with OS and DFS, suggesting that immunoscore may be new prognostic and predictive marker for CRC.

Since the beginning of the 20th century, the immune infiltration of cancer has been suspected as a positive factor in the prognosis of patients.^28^ In several previous studies, the protective role of T‐cell subsets on tumor progression has been consistently reported. Most of the studies have demonstrated that dense infiltration of CD3^+^ and CD8^+^ lymphocytes is associated with less aggressive clinicopathological features and a better prognosis. However, these conclusions have no significant impact on both cancer classification and clinical decision making. Since then, increasing studies have assessed the association between immunoscore and CRC prognosis. However, the potential association between immunoscore and CRC prognosis remains controversial. So, we were interested in the influence of immunoscore on therapy outcomes of OS and DFS in CRC. The study showed that low immunoscore CRCs were related to a 74% reduction in OS and 82% reduction in DFS, compared with high immunoscore CRCs. These findings supported that TILs play an useful role in boosting anti‐tumor immunity against CRC.^4^, ^29^

Subgroup analysis stratified by geographic region showed that the low immunoscore was significantly correlated with poor OS for Europeans, but not significantly correlated with Asians. The results suggest that the effect of immunoscore for CRC prognosis may have a ethnic difference. Considering that the results of the Asian population study are only from two studies, future studies are encouraged to investigate the difference in survival between different races in CRC.

Clinical stage at diagnosis is the most important prognostic factor for CRC.^30^ It is also a prerequisite for identifying CRC patients who are candidates for chemoradiotherapy prior to surgery. Some studies have shown that the immunoscore method was better than the current TNM staging system, but the evidence is restricted to I‐III stages. The subgroup analysis from our study stratified by clinical stage showed that the low immunoscore was much more significantly correlated with poor OS for IV stage patients (HR = 3.61) than for I‐III stage patients (HR = 1.65). However, the pooled HR for IV stage patients just from two studies. So, future studies should therefore be encouraged to be accurately stratified by clinical stage when comparing survival outcomes.

We noticed that one study in our meta‐analysis included a half number of all patients^26^ and seems to be convincing. However, the study has two limitations: (a) The study population was restricted to I‐III stages and did not cover the IV stage of cancer. (b) The cancer site of the study was restricted to colon cancer and not covered rectal cancer. In the included studies, two studies^22^, ^25^ conducted in stage IV patients. So we conducted the meta‐analysis to assess the effects of immunoscore on OS and DFS in CRC patients stratified by clinical stage.

The strengths of the present study include the following: (a) The present analysis is the first to investigate the effects of immunoscore on OS in CRCs; (b) it included a large sample size (4689 CRC cases); (c) to minimize the effect of potential confounders, a strict inclusion criterion, advanced meta‐analysis of HR for survival, and fully outcomes of interest (OS and DFS) were adopted; (d) subgroup analyses stratified by the geographic region, number of patients, max follow‐up time, clinical stage, pathological type, and HR Thus, the effect of potential confounders was minimized; and (e) the results of sensitivity analysis and no publication bias were observed in the analyses, indicating the results are robust.

However, our meta‐analysis has some limitations, including (a) Significant heterogeneity was found among studies when pooling the HRs for OS. To solve this problem, the meta‐analysis of random‐effects model was used to pool data whenever significant heterogeneity was noted. Appropriate well‐motivated inclusion criteria were used to maximize homogeneity, and sensitivity analysis was performed to investigate potential sources of heterogeneity; (b) in this meta‐analysis, the studies selected were limited to English papers and three universal used databases, which may lead to language and selective bias; and (c) subgroup analysis was limited for less studies included, especially for PFS.

In summary, the findings of our meta‐analysis highlight the performance of the immunoscore in predicting the clinical behavior of patients. It is time to begin evaluating immunological markers in international multicenter studies.

## CONFLICT OF INTEREST

No potential conflicts of interest were disclosed.
